# A sewing needle in contact with the cervical dura mater and vertebral artery: A case report: Erratum

**DOI:** 10.1097/MD.0000000000006797

**Published:** 2017-04-21

In the article, “A sewing needle in contact with the cervical dura mater and vertebral artery: A case report”,^[1]^ which appeared in Volume 95, Issue 52, Dr. Fumihiro Arizumi appeared incorrectly as Arizumi Fumihiro. Dr. Shinichi Inoue appeared incorrectly as Inoue Shinichi. Dr. Toshiya Tachibana appeared incorrectly as Tachibana Toshiya. Dr. Keishi Maruo appeared incorrectly as Maruo Keishi. Dr. Shinichi Yoshiya appeared incorrectly as Yoshiya Shinichi.
